# The effect of virtual reality distraction on reducing patients' anxiety before coronary angiography: a randomized clinical trial study

**DOI:** 10.1186/s43044-021-00224-y

**Published:** 2021-11-04

**Authors:** Mostafa Keshvari, Mohammad Reza Yeganeh, Ezzat Paryad, Zahra Atrkar Roushan, Moluk Pouralizadeh

**Affiliations:** 1 Department of Nursing, Shahid Beheshti School of Nursing and Midwifery, Guilan University of Medical Sciences, Rasht, Islamic Republic of Iran; 2 Department of Biostatistics, Medical School, Guilan University of Medical Sciences, Rasht, Islamic Republic of Iran; 3 Shahid Dr. Beheshti Nursing and Midwifery School, Hamidyan suburb,Shahid Beheshti Ave., Guilan, Rasht, Islamic Republic of Iran

**Keywords:** Virtual reality, Angiography, Anxiety state, Vital signs, Heart diseases

## Abstract

**Background:**

Coronary angiography is used as a qualified method to diagnose coronary heart disease. However, patients undergoing coronary angiography experience a great deal of anxiety. The present study is aimed at investigating the effect of virtual reality on anxiety before coronary angiography. In a randomized controlled trial, 60 candidates for coronary angiography were randomly assigned to two intervention and control groups from April to July 2019. Data were collected by Spielberger’s situational anxiety questionnaire. The participants’ anxiety level and their heart rate, respiratory rate, and blood pressure were measured before and immediately after the intervention. The Intervention group received virtual reality intervention, and the control group was cared for based on the hospital routine. Data were entered into the SPSS version 24.0 software (SPSS Inc.) and analyzed using Chi-square, Paired samples, and independent sample *t* tests.

**Results:**

The majority of participants were male (71.25%) and the Mean ± SD age of them in the intervention and control groups was 50.95 ± 4.120 and 52.08 ± 4.002 years, respectively. The mean score of anxiety (*p* < 0.01), heart rate (*p* = 0.001), and systolic blood pressure (*p* = 0.016) after the intervention in the intervention group decreased significantly.

**Conclusions:**

This study indicated the implementation of a VR distraction protocol in the patients could effectively reduce perioperative anxiety and its indices. It showed that VR is a safe method without any complications related to the device and with good acceptability.

*Registration code* IRCT201 40515017693N3.

## Background

Nowadays coronary heart disease (CHD) is one of the most common non-communicable diseases worldwide [1]. It is the largest cause of death in many developed countries. However, the majority of the deaths due to CHD occurred in developing countries with a mortality rate of 220.8 cases per 100,000 people [2]. In Iran in a cohort study, high incidence rates were reported for cardiovascular disease mortality in both men and women [3].

Coronary artery angiography is used as a qualified method to diagnose CHD. It is also a gold standard technology to find the most appropriate treatment method [4]. However, angiography is an important cause of anxiety in cardiac patients, and 82% of patients undergoing angiography experience preoperative anxiety [5, 6]. A high percentage of patients with anxiety develop chest pain [7]. Anxiety can increase the risk of coronary artery spasm, tachycardia, hypertension, and cardiac dysrhythmias. According to the studies, severe anxiety is associated with decreased immunity and cardiovascular dysfunctions [6, 8, 9]. Moreover, because an anxious patient is less likely to collaborate with health care providers, technical problems might occur during coronary artery angiography [10].

Previous studies have shown that patients undergoing cardiac angiography experience a great deal of anxiety. Moreover, these patients receive inadequate and inappropriate care [11, 12]. Different interventions before the angiography can increase the patient's tolerability to procedural discomforts. As one of the major interventions of nurses is to relieve patients’ discomfort, therefore they should assess and control the anxiety using noninvasive methods [5].

Distraction by Virtual Reality (VR) is an effective non-invasive technique that reduces the physical and psychological discomforts in patients. The term *VR* was popularized in the late 1980s by Jaron Lanier, one of the pioneers of the field [13]. The Encyclopaedia Britannica defined VR as “the use of computer modeling and simulation that enables the person to interact with an artificial three-dimensional (3-D) visual or another sensory environment” [14]. Some studies indicated VR is a valuable new technology that provides a better mental preparedness for adults and pediatrics that undergoing invasive procedures using distraction techniques [15–19]. However, the other studies showed contradictory results about the effectiveness of VR on patients’ anxiety [17, 20, 21]. Certainly, the information about the effectiveness of VR on the psychosocial status of patients waiting for coronary artery angiography is scarce. Considering the importance of reducing anxiety in patients with CHD, administrating interventions such as VR might be effective in improving anxiety. Previous studies have demonstrated the application of VR to reduce anxiety in numerous medical settings such as dental hygiene clinics [22], bone marrow aspiration in an outpatient cancer center [22], hand injury requiring surgical wound care [23]. However, the review of the literature revealed that so far no study has investigated the effects of VR on the anxiety of the patients before coronary artery angiography in Iran.

## Objective

The present study is aimed at investigating the effect of virtual reality on anxiety before coronary angiography in patients referred to the cardiovascular, Medical, and Research Center of Guilan University of Medical Sciences.

## Methods

### Trial design

This was a single-center, randomized, controlled trial on the efficacy of VR on anxiety levels in patients candidates for coronary artery angiography. The CONSORT guidelines and the checklist was used for reporting this trial and the description of the interventions. The study contained one intervention and a control group and followed a pretest–posttest design. Each group consisted of 40 patients.

### Study setting

The study was conducted at the school of nursing and midwifery, Guilan University of Medical Sciences, Rasht, Iran. Patients were enrolled from April to July 2019 in Heshmat hospital that is the central cardiac hospital at Guilan University of Medical Sciences.

### Participants

Patients candidates for diagnostic coronary artery angiography through radial artery participated in this study. Eighty participants meeting the inclusion criteria were randomly assigned to one of two groups intervention (VR) and control. The participants were recruited through the angiography department in the Heshmat Cardiac Disease Hospital that was an outpatient setting. Inclusion criteria included the tendency to participate in the study, the first experience of angiography, patients without a history of neurological impairment (such as motor, visual and auditory disabilities), psychological disorders, and taking sedatives. Patients receiving psychological drugs were not included in the study. Also, the candidates were excluded if they had suffered from an inability to tolerate the virtual reality and the occurrence of anxiety symptoms such as fear of closed space. The eligible patients were identified and informed about the study objectives. After giving their preliminary informed written consent to participating in the study, the candidates were offered detailed information and had the opportunity to ask the questions of the study instrument.

### Instruments

To collect the data, a personal information form and the short form of Spielberger’s situational anxiety questionnaire were used.

#### Personal information form

The Personal Information Form included two parts. The first part was demographic characteristics included seven questions: patient's age, gender, marital status, educational level, job title, life place, and having an experience of virtual reality. The second part included clinical indices of blood pressure, respiratory rate, and heart rate.

#### Short form of Spielberger state-trait anxiety inventory (STAI)

The short form of Spielberger State-Trait Anxiety Inventory (STAI) was used to measure the level of anxiety before and after angiography [24]. This Inventory is a tool used to determine the level of state-trait anxiety and included 6-items. The STAI assesses what is felt at the time of measurement. The scoring of this scale is on a *four*-point *Likert* as follows: (1) very low, (2) low, (3) high (4) very high. The range of anxiety scores is between 6 and 20. A higher score indicates greater anxiety. In the current study, Cronbach's alpha coefficient of the scale was found to be 0.80 for pre-angiography and 0.82 for post-angiography.

### Sample size

Sample size calculation was based on the anxiety scores in a similar study [25]. In the study, the mean and standard deviation of anxiety score in the intervention and control group were, respectively, 9.70 ± 3.75 and 11.43 ± 4.51. Considering an alpha of 0.05, a power of 90%, *β* = 0.10 and *d* = 3, sample size was 40 patients per group; a total of 80 participants.

### Randomization method and allocation concealment

**Fig. 1:**
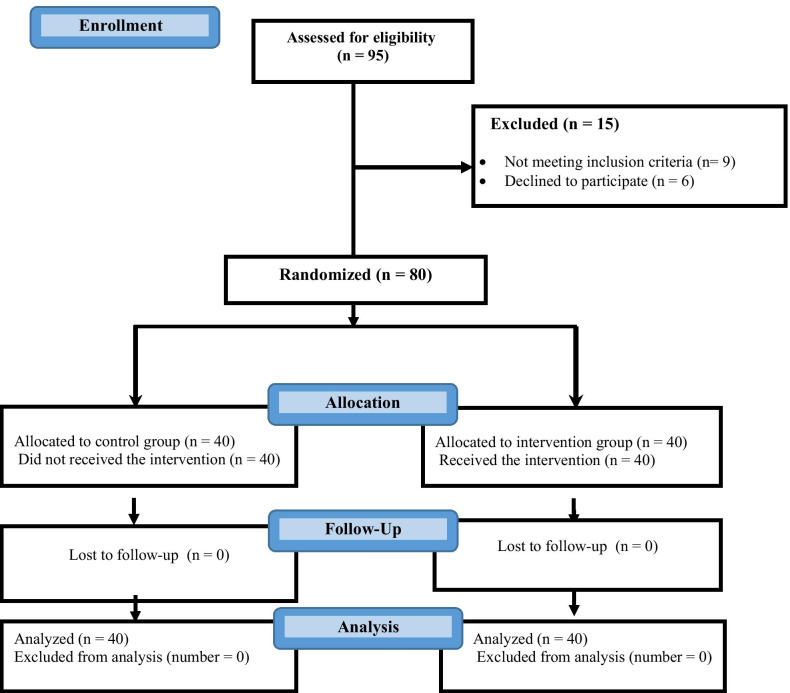
CONSORT flow diagram

Each patient scheduled for angiography who meets our inclusion criteria was asked to participate in the study. Then the participants were asked to fill out the demographic questionnaire. We recorded the number of patients who refused to participate in the study (Fig. 1).

Participants were randomly assigned to one of the two groups of intervention (VR) or control (no intervention). Randomization was carried out using the block method. Blocks of four were used to maintain proportional allocation between the experimental and the control group throughout the study. The randomization list was created using specific software: whoever assigned the participants to the experimental and/or control groups did not take part in the creation of the randomization list and wasn’t aware of its contents. For the randomization and assigning of the participants to the experimental and control groups, we used opaque envelopes. The envelopes were only opened by the nurse carrying out the intervention, after receiving consent from the participants involved in the study.

### Intervention and procedure

Sixty candidates for coronary artery angiography from the cardiac center of Heshmat hospital in Rasht city (A northern city in Iran) took part in this study in two intervention and control groups (30 participants in each group). First, the participants were assessed in terms of the eligibility criteria. Next, the participants were instructed to study objectives and informed consent was obtained. 10 min before the start of coronary artery angiography, the first pre-intervention assessment was carried out. The anxiety level of the participants using STAI as well as their vital signs including; heart rate, respiratory rate, and blood pressure were measured as objective measures of anxiety immediately before the intervention in the two study groups. Blood pressure was measured using an Easy Life hand-held sphygmomanometer in a supine, right-hand position. Heart rate and respiration rate were measured with a digital watch made by *CASIO*. Then the intervention was done for the participants in the intervention group.

Before the start of the angiography, the subjects viewed a 5-min natural scene that was filmed at various natural locations and landscapes such as the beach, mountains, waterfalls, rivers with pleasant sounds by a VR camera. The sounds included soft music, birdsong, and waterfall sounds. A virtual reality video headset made by Remix company which had the ability to change the 360-degree angle of view at the same time as changing the position of the patient's head was used for playing VR video. Also, a Huawei mobile phone and a headphone were used to play the distraction music concurrent with virtual reality film play-back. At the end of the 5-min VR session, subjects were asked to fill out the STAI again and the vital signs were assessed for the second time. The intervention was done only in the intervention group in a private silent room. No intervention and placebo were used for patients of the control group. They completed the study questionnaire at similar times as the intervention group.

### Data analysis

Data were analyzed using SPSS version 24.0 software (SPSS Inc.). Chi-square test was used to assess demographic differences between the two groups. Paired samples and independent sample *t* tests were used to compare the level of state anxiety. The statistical significance was set to 0.05.

### Ethical consideration

This study was approved by the Ethics Committee of Guilan University of Medical Sciences, Rasht, Iran, with the ethical code of IR.GUMS.REC.1397.369. In addition, the study was registered in the Iranian Registry of Clinical Trials with the code of IRCT20140515017693N3. Written informed consent forms were signed by all participants as well as the aim of the study was described to all participants. Moreover, they were ensured that the study was voluntary and they could withdraw from the study at any time.

## Results

We evaluated a total of 95 candidates of coronary artery angiography for eligibility, of whom 15 patients (15.8%) failed to meet inclusion criteria. The excluded patients were ineligible due to respiratory problems (33.3%), chest pain (40%), being prone to vomiting (6.7%), being too debilitated (13.3%), and injury to face (6.7%). Finally, 40 patients were allocated to each group. The participant flow is present in (Fig. 1).

The majority of participants were male (71.25%) and married (76.25%). The educational level of the majority of participants was diploma (30%). The Mean ± SD age of them in the intervention and control groups was 50.95 ± 4.120 and 52.08 ± 4.002 years, respectively. The results of the Chi-square and independent *t* test revealed no significant differences among the two groups with regard to demographic characteristics (Table 1).

**Table 1:** The demographic characteristics of the participants in the studied groups

Variables	Groups		*p* value
	Intervention No (%)	Control No (%)	
Sex			
Male	32 (80)	25 (62.5)	0.083*
Female	8 (20)	15 (37.5)	
Marital statues			
Single	7 (17.5)	5 (12.5)	0.233*
Married	29 (72.5)	32 (80)	
Divorced	3 (10)	3 (7.5)	
Educational level			
Illiterate	1 (2.5)	3 (7.5)	0.611*
Under diploma	12 (30)	10 (25)	
Diploma	12 (30)	12 (30)	
Associate degree	6 (15)	4 (10)	
Baccalaureate	6 (15)	10 (25)	
Master’s degree and higher	3 (7.5)	1 (2.5)	
Occupation			
Worker	6(15)	9 (22.5)	0.626*
Officeholder	8 (20)	7 (17.5)	
Self-employment	15 (37.5)	18 (45)	
Housekeeper	10 (25)	5 (12.5)	
Retired	1 (2.5)	1 (2.5)	
Age Mean ± SD	4.002 (52.08)	4.120 (50.95)	0.219**
Place of residence			
Urban area	67.5 (27)	77.5 (31)	0.317*
Rural area	32.5 (13)	22.5(9)	
Type of insurance			
Health insurance	3 (7.5)	5 (12.5)	0.359*
Social security insurance	21 (52.5)	26 (65)	
Rural health insurance	13 (32.5)	8 (20)	
Army insurance	3 (7.5)	1 (2.5)	
Without insurance	0(0)	0 (0)	
Complementary insurance			
Yes	2 (5)	4 (10)	0.396*
No	38 (95)	36 (90)	
Experience of using virtual reality			
Yes	0 (0)	0 (0)	–
No	40 (100)	40 (100)	

*Chi-square test

**Independent *t* test

The majority of the patients had no history of heart disease (86.25%) and 76.25% were candidates for angiography due to chest pain. There were no significant differences among the two groups in the clinical characteristics and the groups were homogenous (Table 2).

**Table 2:** The clinical characteristics of the participants in the study groups

Variables	Groups		*p* value
	Intervention *N* (%)	Control *N* (%)	
History of cardiac diseases			
Yes	15 (6)	12.5 (5)	0.745*
No	85 (34)	87.5 (35)	
Reasons for referral			
Chest pain	75 (30)	77.5 (31)	0.841 *
Arrhythmia	10 (4)	10 (4)	
Tachycardia	7.5 (3)	5 (2)	
Abnormal ECG	5 (2)	7.5 (3)	
Other factors	2.5 (1)	0 (0)	
Type of admission			
Elective	75 (30)	65 (26)	0.329*
Emergency referral	25 (10)	35 (14)	
Days of hospitalization Mean ± SD	1.297 (2.60)	1.392 (2.90)	0.322**

*Chi-square test

**Independent *t* test

At baseline, no significant differences were reported regarding anxiety scores between the two groups. However, the comparison of the anxiety scores after the intervention revealed a statistically significant difference in the two groups (*p* = 0.002). There also was a significant decrease in intergroup anxiety score (*p* < 0.01) in the intervention group after the intervention (Table 3).

**Table 3:** Scores of situational anxiety in the intervention and control group at baseline and after intervention

	Intervention group	Control group	* *p* value
Before intervention (baseline)	3.144 (14.9)	3.233 (14.4)	0.507*
After intervention	2.131 (13.07)	3.529 (15.1)	0.002*
** *p* value	0.010**	0.292**	

*Independent samples *T* test

**Paired *t* test

A statistically significant difference was revealed in the mean heart rate after the intervention among the two groups (*p* = 0.035). In the intervention group, the paired *t* test showed a significant decrease in the mean heart rate (*p* = 0.001) and systolic blood pressure (*p* = 0.016) from pre- to post-intervention (Table 4).

**Table 4:** Comparison of mean values of vital sign before and after the intervention in the two study groups

	Intervention group	Control group	*p value
Heart rate (min)			
Before intervention	(5.334) 71.6	7.21 (4.562)	0.736
After intervention	69.43 (4.230)	7.4 (4.224)	0.035
**p value	0.001	0.362	
Respiratory rate (min)			
Before intervention	18.93 (0.997)	18.58 (1.466)	0.216
After intervention	18.68 (0.829)	18.53 (1.396)	0.561
**p value	0.115	0.728	
Systolic blood pressure (mmHg)			
Before intervention	135.63 (20.730)	130 (20.646)	0.238
After intervention	(18.115) 125.25	12/ (20.121)	0.522
**p value	0.016	0.677	
Diastolic blood pressure			
Before intervention	79 (9.001)	78.2 (8.883)	0.709
After intervention	(10.025) 76.63	(10.252) 77.3	0.742
**p value	0.235	0.688	

*Independent samples *T*-test

**Paired *t*-test

## Discussion

To the best of our knowledge, this is the first study to investigate the effect of VR on reducing anxiety in candidates for coronary angiography in Iran. The findings revealed virtual reality distraction is effective to reduce anxiety before coronary artery angiography. There was a significant difference between the two groups regarding the mean score of anxiety after the intervention. Also, the reduction from baseline to post-intervention anxiety for the VR group was significantly greater than in the control group.

These results are consistent with other research that used VR to reduce anxiety in patients undergoing coronary artery angiography and showed that the level of anxiety in the patients decreased significantly after the intervention [25, 26]. Morgan et al. demonstrated that an immersive VR experience improves patient familiarity with cardiac catheterization and can reduce pre-procedural anxiety [26]. It seems that VR can divert the patients’ attention with attractive audiovisual materials, which dispels the patient’s anxiety before coronary angiography.

The study of Tadayonfar et al. indicated auditory guided imagery can effectively reduce the level of anxiety in hospitalized patients before cardiac angiography [27]. Guided imagery has been defined as a directed, deliberate daydream that uses all senses to create a state of relaxation and well-being. It is similar to VR and can diminish the anxiety and pain in patients [28]. Considering the similarity of guided imagery with VR, it can be acknowledged that the results are consistent with the present study.

Although researchers have encountered few studies about the effect of VR on pre-angiographic anxiety, there are several similar studies in other fields about the effectiveness of VR and the various audio-visual interventions which are the main mechanism of VR. In this regard, there are studies that are either similar or inconsistent with the findings of the present study.

Brown and Foronda introduced VR as a novel intervention that can reduce perioperative anxiety for patients undergoing surgical and non-surgical procedures [23]. Ganry et al. showed that VR is an effective complementary technique to manage preoperative stress as it allows patients to be immersed in a relaxing environment and is a safe way to reduce stress levels [29].

Some studies in other fields also reported a positive effect of virtual reality in the reduction of anxiety and other psychological symptoms such as stress and depression in patients with cancer [30] and the endoscopic candidates [31].

Contrary to the results of the current study, previous research in china among patients with coronary artery disease has not reported a reduction in anxiety following an auditory and sensory stimulation which was similar to the VR [32]. It seems that the inconsistency of the findings is due to the lack of use of visual stimulation.

The results of the present study indicated a significant reduction in the heart rate and blood pressure after the intervention in the intervention group in comparison with the control group.

The results of the other study that investigated the effect of an auditory relaxation technique on anxiety and physiologic indices in patients before angiography showed a significant decrease in the anxiety score, heart rate, and blood pressure in the intervention group [32] that is consistent with our findings. Decreased blood pressure after relaxation and distraction is caused by muscle relaxation, decreased vascular resistance, and decreased sympathetic system activity in patients.

This study is the first research to examine the effect of virtual reality exposure in candidates of coronary artery angiography in Iran. In order to improve the generalizability of the results, we recommend more studies for the development of evidence-based practice.

There are some strengths and limitations of the study. One of the strengths of this study was that it consisted of two groups. However, the short duration of the intervention and the follow-up were the limitations of this study. Similar to the other study and innovations in the field of facilitators of non-invasive care and treatment [33], this study was conducted for the first time in Iran and the impact of virtual reality on the pre-angiography anxiety was confirmed as an innovation.

## Conclusions

The results of this study indicated a significant difference between the two groups with respect to the mean score of anxiety, heart rate, and blood pressure after the intervention. Implementation of the VR distraction protocol as a practical non-invasive method could effectively reduce pre-angiography anxiety and its indices in the angiography candidates. Further studies are needed to more accurately assess its long-term effects in reducing anxiety and complications caused by angiography.

## Data Availability

Not applicable.

## Abbreviations

CHD: Coronary heart disease
VR: Virtual reality
STAI: Short form of Spielberger state-trait anxiety inventory

## References

[1] Qin S, Gu Y, Song T (2019). Effect of peer support on patient anxiety during the coronary angiography or percutaneous coronary intervention perioperative period: a protocol for a systematic review and meta-analysis of randomised controlled trials. BMJ Open.

[2] Benjamin EJ, Blaha MJ, Chiuve SE, Cushman M, Das SR, Deo R (2017). Heart disease and stroke statistics-2017 update: a report from the American Heart Association. Circulation.

[3] Talaei M, Sarrafzadegan N, Sadeghi M, Oveisgharan S, Marshall T, Thomas GN et al (2013) Incidence of cardiovascular diseases in an Iranian population: the Isfahan Cohort Study. Arch Iran Med 16(3):0 23432164

[4] Nekouei ZK, Yousefy A, Manshaee G, Nikneshan S (2011). Comparing anxiety in cardiac patients candidate for angiography with normal population. ARYA Atherosclerosis.

[5] Moradi T, Adib HM (2015). State and trait anxiety in patients undergoing coronary angiography. Int J Hosp Res.

[6] Habibzadeh H, Milan ZD, Radfar M, Alilu L, Cund A (2018). Effects of peer-facilitated, video-based and combined peer-and-video education on anxiety among patients undergoing coronary angiography: randomised controlled trial. Sultan Qaboos Univ Med J.

[7] Assari S, Zandi H, Ahmadi K, Saleh DK (2017). Extent of coronary stenosis and anxiety symptoms among patients undergoing coronary angiography. J Tehran Univ Heart Center.

[8] Asgari MR, Barari L, Ghorbani R, Darabiyan M, Eskandarian R, Ghods AA (2019). Anxiety levels in patients candidate for coronary artery angiography. Koomesh.

[9] Gu G, Zhou Y, Zhang Y, Cui W (2016). Increased prevalence of anxiety and depression symptoms in patients with coronary artery disease before and after percutaneous coronary intervention treatment. BMC Psychiatry.

[10] Aboalizm SE, El Gahsh NF, Masry SE (2016). Effect of early nursing preparation on anxiety among patients undergoing cardiac catheterization. Am J Nurs.

[11] Jamshidi N, Abaszade A, Najafi-Kaliani M (2012) Stress, anxiety and depression of patients before coronary angiography. Zahedan J Res Med Sci (Tabib-e-Shargh) 13(1) (**Persian**)

[12] Vardanjani MM, Alavi NM, Razavi NS, Aghajani M, Azizi-Fini E, Vaghefi SM (2013). A randomized-controlled trial examining the effects of reflexology on anxiety of patients undergoing coronary angiography. Nurs Midwif Stud.

[13] Riener R, Harders M (2012) Introduction to virtual reality in medicine. In: Virtual reality in medicine. Springer, London

[14] Tytler J (2020) Encyclopædia Britannica 2020 January 07, 2020. https://www.britannica.com/biography/James-Tytler

[15] Arjmandnia A, Maleki F, Sharifi H, Heydari K (2017). Investigating the effectiveness of physical education program on improving the social competence of mentally retarded students in the pre-vocational course. Except Educ.

[16] Bohil CJ, Alicea B, Biocca FA (2011). Virtual reality in neuroscience research and therapy. Nat Rev Neurosci.

[17] Noben L, Goossens SMTA, Truijens SEM, Van Berckel MMG, Perquin CW, Slooter GD (2019). A virtual reality video to improve information provision and reduce anxiety before cesarean delivery: randomized controlled trial. JMIR Mental Health.

[18] Dahlquist LM, Weiss KE, Dillinger Clendaniel L, Law EF, Ackerman CS, McKenna KD (2008). Effects of videogame distraction using a virtual reality type head-mounted display helmet on cold pressor pain in children. J Pediatr Psychol.

[19] Frere CL, Crout R, Yorty J, McNeil DW (2001). Effects of audiovisual distraction during dental prophylaxis. J Am Dental Assoc.

[20] Umezawa S, Higurashi T, Uchiyama S, Sakai E, Ohkubo H, Endo H (2015). Visual distraction alone for the improvement of colonoscopy-related pain and satisfaction. World J Gastroenterol WJG.

[21] Kerimoglu B, Neuman A, Paul J, Stefanov DG, Twersky R (2013). Anesthesia induction using video glasses as a distraction tool for the management of preoperative anxiety in children. Anesth Analg.

[22] Alshatrat SM, Alotaibi R, Sirois M, Malkawi Z (2019). The use of immersive virtual reality for pain control during periodontal scaling and root planing procedures in dental hygiene clinic. Int J Dental Hygiene.

[23] Brown K, Foronda C (2020). Use of virtual reality to reduce anxiety and pain of adults undergoing outpatient procedures. Informatics.

[24] Marteau TM, Bekker H (1992). The development of a six-item short-form of the state scale of the Spielberger State—Trait Anxiety Inventory (STAI). Br J Clin Psychol.

[25] Bagheri-Nesami M, Shorofi SA, Zargar N, Sohrabi M, Gholipour-Baradari A, Khalilian A (2014). The effects of foot reflexology massage on anxiety in patients following coronary artery bypass graft surgery: a randomized controlled trial. Complement Ther Clin Pract.

[26] Morgan H, Gallagher SM (2019). 59 The effect of a virtual reality immersive experience upon anxiety levels in patients undergoing cardiac catheterisation: the virtual cath trial. Heart.

[27] Tadayonfar M, Foji S, Mohsenpour M, RakhshaniI M (2014). The effect of guided imagery on patients' anxiety undergoing cardiac catheterization. J Sabzevar Univ Med Sci.

[28] Gonzales EA, Ledesma RJ, McAllister DJ, Perry SM, Dyer CA, Maye JP (2010). Effects of guided imagery on postoperative outcomes in patients undergoing same-day surgical procedures: a randomized, single-blind study. AANA J.

[29] Ganry L, Hersant B, Sidahmed-Mezi M, Dhonneur G, Meningaud J (2018). Using virtual reality to control preoperative anxiety in ambulatory surgery patients: a pilot study in maxillofacial and plastic surgery. J Stomatol Oral Maxillofac Surg.

[30] Aliakbari M, Alipor A, Ebrahimimoghadam H, Fekraty M (2017). The effect of virtual reality (VR) on psychological disorders in cancer caseses. Military Caring Sci.

[31] Heidari M, Shahbazi S (2013). Effect of Quran and music on anxiety in patients during endoscopy. Knowl Health.

[32] Taylor-Piliae RE, Chair S-Y (2002). The effect of nursing interventions utilizing music therapy or sensory information on Chinese patients’ anxiety prior to cardiac catheterization: a pilot study. Eur J Cardiovasc Nurs.

[33] Valsecchi O, Vassileva A, Cereda AF, Canova P, Satogami K, Fiocca L (2018). Early clinical experience with right and left distal transradial access in the anatomical snuffbox in 52 consecutive patients. J Invasive Cardiol.

